# Wheel of Wellbeing (WoW) health promotion program: Australian participants report on their experiences and impacts

**DOI:** 10.1186/s12889-021-12076-x

**Published:** 2021-11-08

**Authors:** Daniel Spain, Victoria Stewart, Helen Betts, Amanda J. Wheeler

**Affiliations:** 1 School of Health Sciences and Social Work, Griffith University, Brisbane, South East Queensland Australia; 2 Menzies Health Institute Queensland, Griffith University, Brisbane, South East Queensland Australia

**Keywords:** Mental wellbeing, Wheel of Wellbeing, Community capacity building, Social capital, Mental health, Health promotion

## Abstract

**Background:**

Community-based mental health promotion programs focus on improving individual and community wellbeing by strengthening resilience and building capacity to support positive health outcomes. The Wheel of Wellbeing (WoW) is an example of such a program, promoting activities that support social engagement and positive emotions within a holistic framework underpinned by positive psychology. WoW is intended to be flexibly implemented in each community, training community members who implement behaviour change activities in their local community, workplace and educational settings.

**Method:**

This study aimed to understand the opinions and experiences of a sample of individuals who had participated in a range of WoW training programs; documenting the impact on participant behaviours and professional practices, and how the WoW framework was subsequently employed within their communities. Using Ripple Effects Mapping evaluation processes to guide a focus group, nine WoW training participants collectively reflected on the program impacts, generating consensus themes and a mind map. Mind map qualitative data were entered into XMIND mapping software and reviewed with the focus group transcription and field notes.

**Results:**

Thematic analysis identified three themes: increased community involvement and engagement (strengthening community connections); improved health, emotions and behaviour (motivating change to health behaviours); and flexible resources which could be utilised in a range of settings (easily incorporated in the existing organisational cultures).

**Conclusions:**

The results of this study support the premise that the WoW framework can be an effective framework for guiding wellbeing promotion activities, with participants championing a ‘ripple effect’ across individual, family, friendship, professional and community networks.

## Background

Mental health includes psychological, social, and emotional wellbeing, and along with physical health it is part of every individual’s life. Poor mental wellbeing is understood as a risk factor for developing a mental health disorder; an estimated 10.7% of the world’s population live with a mental health disorder, accounting for 13% of the global burden of disease [1, 2]. In addition to the impact of mental illness(es) on individuals, there are significant societal costs such as those arising from treatment (e.g., hospitalisation, medication) and lost productivity (e.g., work absences, unemployment) [3, 4]. It is partly for these reasons that the World Health Organisation (2018) identified the promotion of wellbeing and mental health as one of the three strategic priorities to improve global health over the next five years (2019–2023).

In addition to the provision of, and access to, evidence based mental health treatment, there are increasing calls for early intervention, preventative community-based approaches and promotion of mental wellbeing [5]. We acknowledge that interventions that promote positive mental wellbeing need to take into account individual, community and structural factors if they are to be effective, with deprivation and inequalities diminishing the individual and community resources needed to generate and maintain good mental health [6]. Social support and engagement in community life have been found to provide protection during times of adversity or stress, with growing evidence to support the effectiveness of community-based mental health prevention and intervention programs [7–9].

Robust mental wellbeing is linked to living a longer, more fulfilling life, better physical health, stronger and more enduring relationships, and other psychological, sociological and economic benefits [10]. This is supported by earlier research which demonstrated that practices such as social connections, keeping physically active and engaging in continuous learning, can improve mood, strengthen relationships, keep populations healthy and reduce the risk of depression [11].

Mental health promotion programs seek to enhance mental wellbeing through several processes including the reduction of sub-syndromal symptoms of mental illness, promoting wellbeing, or enhancing resilience through changing individual and community behaviours [12]. Community capacity building involves the use of community action to improve and promote the health of community members and is an essential element in the maintenance of health promotion and prevention programs [13, 14].

### The Wheel of Wellbeing

The Wheel of Wellbeing (WoW) is one such health promotion program that has been developed to promote the wellbeing of individuals and communities [11]. WoW was initially developed in 2009 as part of the *Well London* program, a city-wide health improvement program [15]. Since then, WoW has been implemented in a number of ways in countries around the world and has a dedicated website with information and downloadable resources [16].

The WoW framework uses a Theory of Change [17] approach to positively promote mental health and wellbeing by focusing interventions on enhancing participants’ experiences of daily life through emphasising social engagement, positive emotions and meaningfulness [15, 18, 19]. WoW supports people to exercise individual and community agency in achieving wellbeing; encouraging and enabling them to behave or act in ways which enhance their overall mental wellbeing [20–22].

**Fig. 1:**
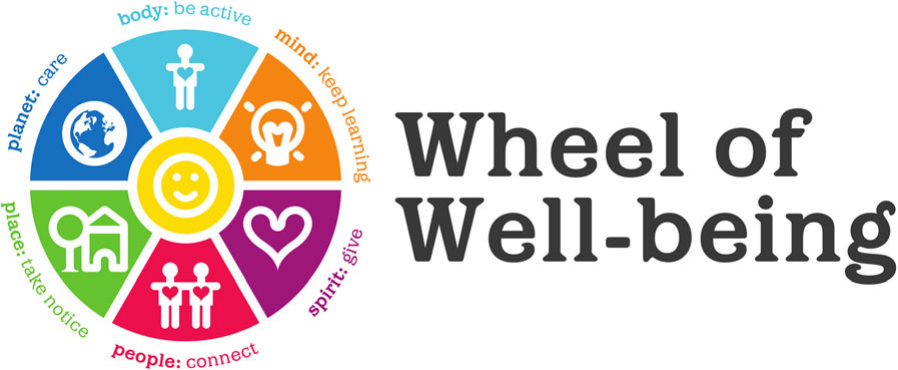
Wheel of Wellbeing, Implemental, https://www.wheelofwellbeing.org/

WoW focuses on positive mental health through the provision of a holistic framework underpinned by the fundamentals of positive psychology [11]. Wo is constructed on six dimensions of wellbeing (Fig. 1) which were based on five evidence-based suggestions regarding actions which promote wellbeing identified in the Foresight review [23]. The six elements of the wheel and examples of associated actions include: i) body (being active), ii) mind (life-long learning and creativity); iii) spirit (giving and positive emotions); iv) people (connecting to others); v) place (neighbourhoods); and, vi) planet (caring for the environment). As such, WoW was developed as a practical and integrated community approach to increasing mental health and wellbeing.

### The WoW training program

Whilst the WoW framework is intended to be used flexibly within each community, the concepts which underpin the framework are typically introduced through a training program. The training program aims to increase community members’ understanding of wellbeing and the science/theory/evidence base for behaviour change; identify relevant activities to improve wellbeing; and stimulate motivation for individual and community engagement in these activities [17]. Resources to supplement the training program and processes to assist with community integration are also provided.

It is intended the knowledge and skills gained from training program/s undertaken by individuals and implemented within their local communities, workplaces and/or educational settings, thus create a flow-on, or domino, affect which escalates and broadens the impact [17]. Training is available at different levels, ranging from introductory/short courses to more intensive and advanced (train-the-trainer) programs.

Whilst the WoW framework has been utilised as a health promotion tool for some years, limited research has been undertaken to understand implementation processes and mechanisms, and whether outcomes have been achieved and/or sustained. However, feedback from previous participants has been generally positive, indicating WoW activities have resulted in improved well-being scores and increases in positive behavioural actions, knowledge, and life satisfaction. To date there appears to have been no research into possible longer-term WoW impacts.

## Methods

This study sought to understand the opinions and experiences of a sample of individuals who had participated in a range of WoW training programs and aimed to document impact on participant behaviours and professional practices. We were also interested in learning how the WoW framework was subsequently employed within participant’s communities.

### Study setting

The WoW framework and training programs were introduced to communities within Queensland, Australia, by the Queensland Mental Health Commission (QMHC) through the pilot ‘Wellbeing Hub’ initiative (Queensland Mental Health Commission, 2016). Wellbeing Hubs were established in three Queensland locations (Logan and Southern Moreton Bay Islands, Central Highlands, Far North Queensland) to review and strengthen the effectiveness of local mental health and wellbeing activity [24]. Training was delivered across Hub sites between July 2017 and November 2019 and targeted individuals, mental health service providers, mental health/community workers and community members with an interest in improving mental health and wellbeing. On completion of training, all participants were provided with one year of access to online WoW resources and activities.

Two trainers provided three levels of WoW training [16] comprising:

1. One day WoW workshop during which participants were introduced to concepts underpinning the WoW framework and participated in wellbeing activities.
2. WoW Practitioner (‘DIY Happiness’) course comprised eight half-day workshops delivered by an advanced WoW trainer. Participants completed a WoW personal learning log, explored the evidence underpinning the six dimensions of the WoW, and participated in WoW activities. Training equipped participants with the skills they needed to facilitate the use of the WoW framework within their local communities.
3. WoW Advanced Trainer (train-the-trainer) course required that participants first undertook the ‘WoW practitioner’ course. After completing reflective journals, learning logs and mentoring sessions, participants were encouraged to deliver training workshops within their local communities.

### Participants

Participants who had completed any of the three levels of WoW training delivered in the Logan and Southern Moreton Bay Islands (SMBI) regions were invited to participate in the study. An email invitation containing research information was sent to all eligible participants identified through the WoW training database. The initial email invitation was followed up by a reminder after four weeks. This study protocol is performed in accordance with the relevant guidelines.

### Study design

Evaluating changes, based on community health-focused programs, at an organisational, group, community or individual level is challenging [25]. Applying the interdependence principle of ecological and systems theory, planned community-based programs can create ripples of change, with many of these ‘ripples’ unappreciated, unobserved or undocumented [26]. Ripple Effects Mapping (REM) is a qualitative participatory evaluation method which assesses mid-term and community level changes resulting from program activities [27, 28]. REM is seen as an appropriate methodology for evaluating change where the program itself is distributed over larger populations and involve a broad range of stakeholders [27, 29]. This methodology was seen as particularly relevant to assist with understanding the impact of WoW which had engaged participants from a range of community, non-government and government organisations.

REM uses a qualitative approach to actively engage stakeholder participation in the developing a visual map to identify the impact of community programs and community interventions. An interactive process utilising a facilitated focus group engages participants to process and reflect, both individually and collectively, through visual mapping techniques [29]. It is an engaging and interactive process that relies on storytelling to develop a deeper understanding of program outcomes. Three discrete methods for facilitating REM sessions have been identified and include web mapping, in-depth rippling, and theming [27]. The theming and rippling approaches were selected for this research, as they allowed participants to reflect on their most significant experiences and knowledge of application of the WoW framework in practice.

There are four core elements to REM evaluation processes including appreciative inquiry (AI), participatory approaches, interactive group interviewing and reflection and mind mapping [27]. AI interviewing supports participant generation of knowledge about the program, while participatory and interactive group processes actively integrate stakeholders into the evaluation process, allowing for the generation of new understandings and perspectives [27]. Collective reflection assists the group identify connections between program impacts which is visually mapped through the mind mapping process [27].

#### Data collection

A focus group of four hours duration was undertaken in February 2020 at a suitable venue at the University campus in Logan Queensland with refreshments provided. Written consent was obtained prior to participation. Participants were asked to provide demographic details including employment status, gender, cultural background and details about any previous completion of a WoW training program. The focus group was co-facilitated by three members of the research team (DS, VS, HB) with photographs of the whiteboard content used in the data analysis. The focus group was recorded and extensive field notes were made by one of the researchers (DS). These were subsequently written up, and along with the transcribed recording were disseminated to all members of research team.

At the commencement of the focus group, participants interviewed each other using AI processes. To assist the AI interview process participants were introduced to the seven community ‘capitals’: social, natural, cultural, human, political, financial and built [30]. Descriptors of these capitals remained on display for participants to refer to throughout the focus group. The concept of community capitals was introduced to encourage participants to think systematically and deeply about the outcomes of WoW. Participants were asked to consider a number of questions pertaining to the particular ways in which the WoW framework, had impacted on their personal, community and work life, what they were now doing differently as a result of participation, and the benefits and/or achievements they and others had experienced. Participants were provided with ‘post-it’ notes to record their thoughts which were then used to facilitate larger group discussions.

The group then engaged in a facilitated discussion, sharing what they had learnt from the AI interviews, inviting all participants to provide further detail regarding their narratives/stories. Details of the stories were collected on a whiteboard and further questions from the focus group facilitators encouraged participants to reflect on their experiences (e.g. *How did that help? How did that impact your community? What has been the impact of doing those things?);* this in-depth examination helped to generate rich narratives.

**Fig. 2:**
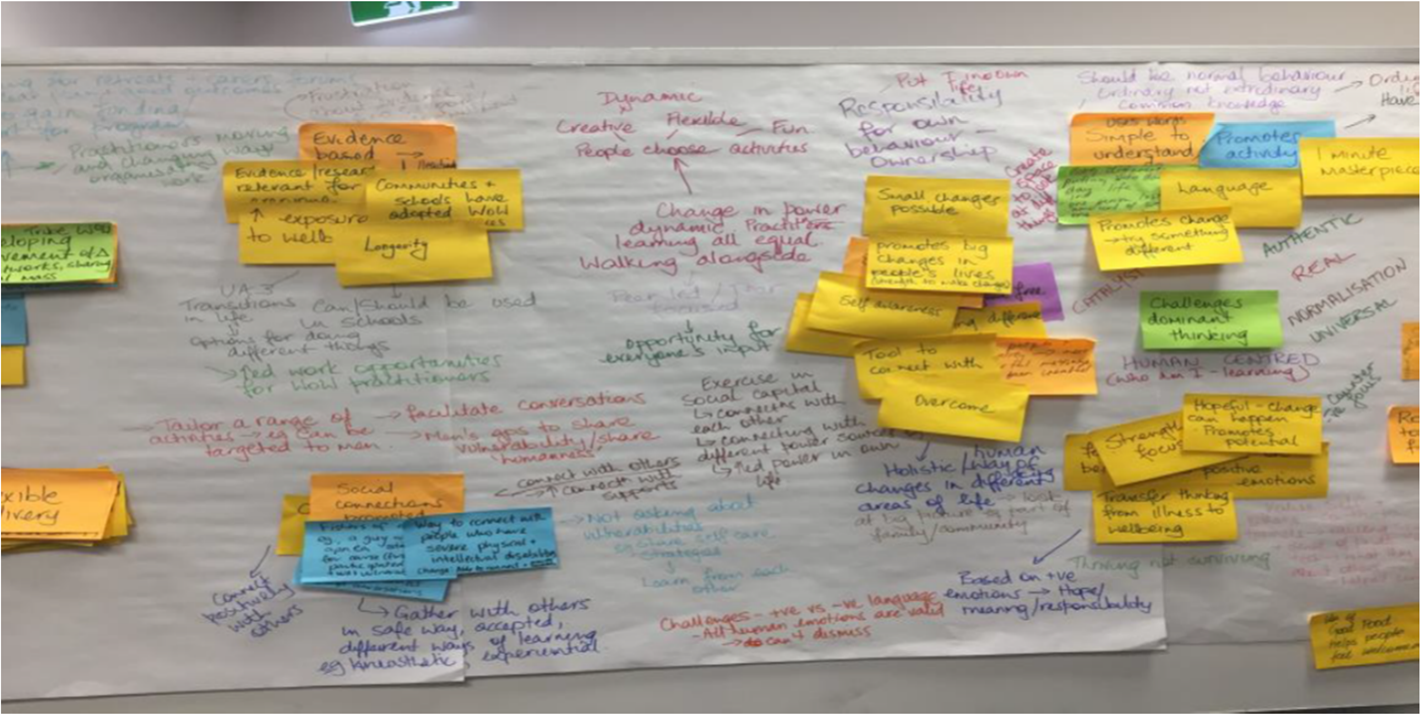
Mind map drawn during REM focus group

Once the stories had been documented, a facilitated theming of the stories was undertaken by the group. Participants reviewed each other’s stories and brainstormed possible themes that could capture the main actions and impacts, before grouping them into broader, common themes. Groupings were discussed with the participants until consensus on final themes was reached and a mind map created (Fig. 2).

### Data analysis

Following the completion of the REM focus group, the qualitative data from the mind map (Fig. 2) were entered into the recommended mapping software XMIND© [27]. Data included the mind map, the researcher’s field notes, and additional stories and information gained from reviewing the recorded content. Data analysis followed the six steps of thematic analysis outlined by Braun [31].

**Fig. 3:**
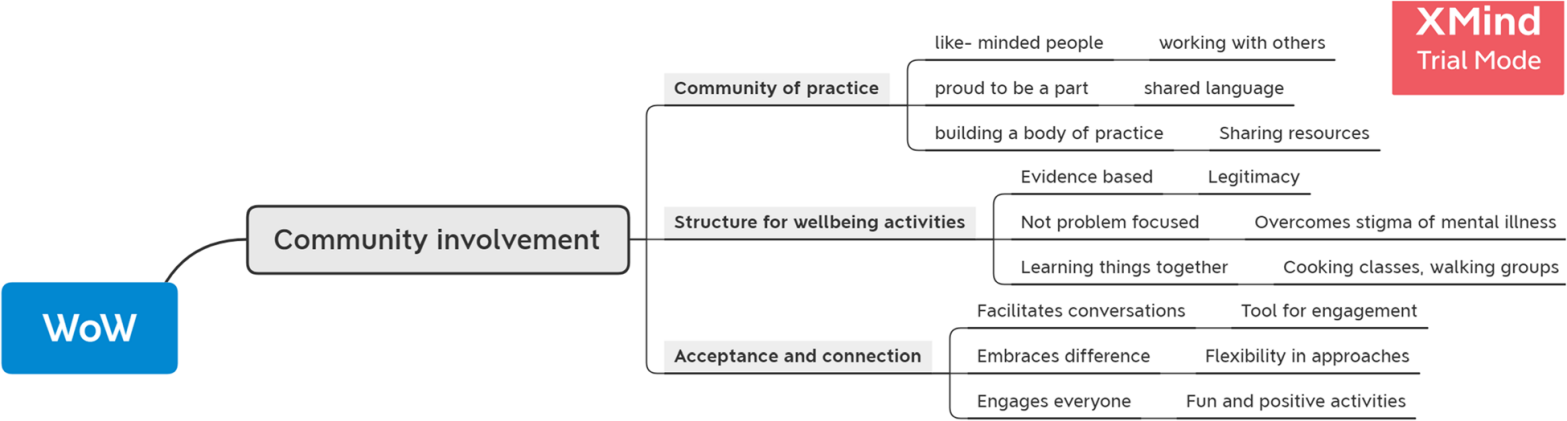
Example of a portion of Mind Map using XMIND software

The first step involved the researchers familiarising themselves with the data set, followed by multiple reviews of the data by two researchers (DS, VS) to ensure the preliminary themes and the mind map did not contain overlapping data and themes were linked coherently. Step three and four involved an iterative process, with both researchers coding transcripts and fieldnotes to both the pre-determined themes identified by the group, and emergent themes from discussions which had not been documented at the time of the focus group. The themes and mind map were reviewed a number of times by the research team until agreement was reached and a coherent mind map (Fig. 3) was produced. This systematic analysis process aimed to improve the credibility, dependability, transferability and confirmability of the results [31, 32].

## Results

Nine people participated in the focus group; seven female and two males. Most identified as Caucasian (*n* = 7) with one participant of Aboriginal or Torres Strait Islander descent and one of Māori descent. All were employed (*n* = 7) or volunteered (*n* = 2) in the community; the majority (*n* = 8), had completed both a 1-day workshop and the Practitioner course. Two participants were certified as advanced WoW practitioners.

Thematic analysis identified three themes (Table 1) describing how participants’ reported on their experiences of WoW.

**Table 1:** Themes and descriptors

Theme	Description	Subthemes
1. Increased community involvement and communication	WoW training builds on skills of groups, individuals and communities to foster new relationships, social connection and collaboration	• Creating a community of practice • Providing an evidence base and structure for wellbeing activities • Facilitating acceptance, connection, and social inclusion
2. Improved health, emotions, and behaviour	Promotes motivation for change toward better health	• Positively promotes new understanding and influences sharing • Facilitates positive conversations • Embraces diversity
3. Increased flexibility across a range of settings	WoW is flexible with changes easily incorporated into existing programs and organisations	• Easily adapted • Changing workplace cultures

Quotes illustrating the themes are provided in the text and Tables 2, 3 and 4 with identifiers (e.g., P1) to distinguish between participants.

**Table 2:** Supporting quotations for Theme 1

1. Increased community involvement and communication	
Creating a community of practice	“I really enjoyed the opportunity to partner with friends and colleagues across organisations to co-deliver stuff and work with all different people collectively across Logan.” (P4) “Having done the [WoW training], it helped me step up and actually say, I can do a new thing, I don’t have to copy what has been done in the past. So, I was doing something new that was still evidence-based to work from … For three years we were funded.” (P5)
Providing an evidence base and structure for wellbeing activities	“It was a chance for people very early in their lives to be exposed to a whole bunch of selfcare, let off a lot of steam and have fun. It was a great organising structure to deliver such a big thing, in quite a strategic way.” (P4)
Facilitating acceptance, connection, and social inclusion	“Since being introduced to WoW, the view I now have is that, WoW doesn’t look at mental illness, it looks at mental health challenges. Mental health is about good thoughts feelings and behavioural outcomes. WoW gives another perspective by looking at when those outcomes are challenged, without giving all focus to the negatives”. (P3) “But with a WoW focus young people can actually not only just address their own problems and their friends problems but they can actually help and address their own and their friends wellbeing, having influence on their friends wellbeing and yeah, leading by example, being role models. By just pushing a little bit of the WoW content, changing the language around and being more wellbeing then problem focused, it’s a powerful message”. (P7)

**Table 3:** Supporting quotations for Theme 2

2. Improved health, emotions and behaviour	
Positively promotes new understanding and influences sharing	“She got really involved because of WoW. Initially she shared her interest in weaving, which she was taught by the Indigenous People on Island and because of the WoW group, they gave her permission to teach other people weaving. So, she got paid for it, she turned up and taught us how to weave. This activity then led to ongoing work and ultimately further study”. (P5) “We had one of the mum’s that came to us she was very, very, overweight. We were going through the wheel and got to the body be active (domain) and she decided that she wanted to lose some weight. Due to her size, she couldn’t participate in some of the stuff she wanted to in day-to-day society. We started the body be active program with her off the back of that. She ended up losing something like 15 kg and still is losing weight …. It is actually such a lovely story and I feel so proud and privileged to have been a part of it.” (P6)
Facilitates positive conversations	“We used the principles learnt through WoW (positive affirmation cards) as an acknowledgement tool for people in the community (i.e. firemen, ambulance, police, women’s centre). They were all so taken back that someone had taken time to actually acknowledge what these people do within the community. This simple act improved relationships between our organisation and these important services.” (P6) “I have incorporated WoW into our yarning circles [an important communication process in Aboriginal and Torres Strait Islander peoples culture]. It really helped men connect with themselves by coming from a place of hope and not fear.” (P8)
Embraces diversity	“Imagine how much could be enhanced if we (the community) had a more sustainable focus on wellbeing, I feel you can use the concept of WoW to tailor positive psychology to any audience.” (P4)

**Table 4:** Supporting quotations for Theme 3

3. Increased flexibility across a range of settings	
Easily adapted	“WoW is a framework that is quite flexible that it can be tailored to each individual group and incorporated into existing programs, through the way it is presented, it is tailored through its flexibility.” (P4)
Changing workplace cultures	“WoW challenges the systems, job descriptions and tasks, a lot of us in this space, it’s a very volatile space for employment. Our (WoW) practitioners are moving to other places and they bring WoW with them, changing the way things are done in organisations. It’s that humanness, connections and the way it (WoW) has profoundly affected us and that we enjoy doing it. It’s not really the structure telling us what to do so much anymore, it’s us trying to tell the structure what to do” (P.5).

### Increased community involvement and communication

The first theme described WoW as a tool which had strengthened community connections.

#### Creating a community of practice

A community of practice is described as a group of people who have a common interest, voluntarily coming together to share ideas and grow their practice [33]. Participants reported that WoW training had been a catalyst in creating a WoW community of practice; engaging groups of like-minded practitioners and individuals within the community sector, fostering connections and bolstering confidence in the promotion of wellbeing. It was reported that since being introduced to WoW, participants had strengthened relationships with others who had an interest in mental wellbeing, sharing knowledge and activities – “instead of feeling like I was alone, I felt like I was part of a team, and I was able to explore other stuff that was interesting around wellbeing.” (P4).

Members of the community of practice reported supporting each other with designing new programs, enhancing existing programs and accessing new sources of funding. WoW skills were reportedly seen positively by employers with one participant reporting that WoW was a factor in their most recent employment – “I was surprised how quickly I was snapped up by this organisation after discussing my WoW activities.” (P3).

#### Providing an evidence base and a structure for wellbeing activities

The WoW training was described as providing participants with increased knowledge in concepts underpinning positive wellbeing and skills to support wellbeing activities and the implementation of community-based programs. Participant confidence gained from the training had resulted in conference presentations and the development of information and journal articles.

#### Facilitating acceptance, connection, and social inclusion

WoW activities were seen as focusing on creating environments of acceptance and establishing a sense of inclusion in a non-judgemental environment. A focus on supporting strengths as well as encouraging all to learn and grow were seen as important aspects of the WoW training and activities. Participants identified that as practitioners, they had facilitated activities that focused on building shared positive experiences which had resulted in positive social connections for all. The importance of activities being “shared not led” (P6) and “peer led and peer focused” (P7) were seen as important in facilitating community connections and contributed to sustainable social connections:

“We found lasting social connections were made, women had met up outside of our group. There was one Mum, and she never went out for a walk ever. In our WoW group we talked about it (walking), so she actually went out for a walk one day and met up with one of the other Mums from the group, they became friends. Then they started meeting the kids at the park and the group continued to grow and I just thought how such a simple thing that has led to such a change for these people.” (P6).

### Improved health, emotion, and behaviour

This second theme described how WoW training promoted motivation for change which encouraged physical and mental health behaviours.

#### Positively promotes new understandings and influences sharing

WoW training provided a platform for building a positive learning environment, encouraging participants to share their knowledge, skills, tools, and strategies. Participants described attendance as generating a “thirst for learning” (P3) and a “sense of achievement” (P6); one participant commented that “WoW is a way of life” (P9). Participants reported that new insights and understandings were accessed through a variety of practical activities such as cooking something new, starting a gardening club, reading a book, re-discovering an old hobby, and/or taking up further education or training. Insights were shared between families and friends, and with the wider community.

The WoW focus on supportive and sharing environments was seen as an important in supporting the adoption of health attitudes and behaviours. The use of short, fun, team-building activities which aimed to increase positive emotions were adopted by many participants in their work with disadvantaged communities. One participant, who had introduced WoW into an existing community program, described how some features had been incorporated “into day-to-day life” (P6) and a focus on the ‘body be active’ dimensions of WoW had resulted in positive health behaviours for community members.

#### Facilitates positive conversations

WoW activities reportedly inspired individual participants to become more engaged with others around them by increasing a focus on positive aspects in their life and encouraging them to become active within their individual social, community lives. WoW activities were seen to shape positive and supportive conversations, with benefits from this flowing on to families and the wider community:

“Since I did the WoW program two and a half, three years ago, huge, huge impacts for me. What I’ve learnt, I’ve thrown off to other people, to help them become more positive people. The impact it made on me as a parent, it did make an impact on me and my children.” (P9).

#### Embraces diversity

The WoW focus on inclusion was highlighted by participants and WoW was acknowledged as a tool that could be used across cultures. Participants explained the similarity between local cultural practices and norms and those espoused by WoW. One participant explained how WoW was similar to their experience of “home teaching, the circle way” (P3), providing a cultural understanding which concentrated on the positive aspects of life:

“I reflect on the WoW and my home teachings and this is my perspective of WoW. I seek not to do what has always been done but try something different, in hope of different results and better outcomes.” (P3).

The stories shared by participants described the resources provided by WoW as diverse tools that could be shared across cultures and had the potential to bring cultures together by: “tapping into, and really acknowledging and celebrating, a whole lot of different cultural perspectives. It brings together what people have in common” (P7).

### Flexible in a range of settings

In this final theme, participants report on their experiences of using WoW resources, viewing them as flexible tools which were readily incorporated into existing programs and organisational cultures.

#### Easily adapted

Participants found that concepts within the WoW framework had been easy to incorporate into their employing organisations/workplaces. Indeed, some had already adopted WoW resources into their in-house programs. Examples of how participants had incorporated WoW into existing frameworks were broad and varied across settings including schools, family programs, carers’ retreats and forums, parenting groups, peer skills programs for young people, yarning circles, mental health settings, University of the 3rd Age (U3A), and drug and alcohol focused programs. WoW activities were also seen as easily adapted to suit a range of settings and ages.

#### Changing workplace cultures

Participants frequently reported that they had introduced new knowledge and skills from WoW into their workplace environments. One participant shared how they had introduced WoW to staff within their education setting, resulting in the introduction of self-care spaces (e.g. a meditation room) and regularly scheduled activities to enhance staff wellbeing:

“I have added WoW as one of the things I do. I run facilitator training for people who work in schools (teachers, teacher aids, administration staff and school nurses).” (P7).

The significant wellbeing impacts reported in educational settings rippled across a number of schools as WOW-based interventions were introduced: “It has helped professionals put in place healthy boundaries within their work setting around the concept of self-care” (P7). Another participant described how the introduction of WoW concepts into their workplace had improved staff meetings, relationships between colleagues, decision making and problem-solving capacities, thereby increasing workplace productivity: “It’s made a huge difference to the workforce and how we connect with each other” (P6).

## Discussion

WoW is a strengths-based, community-driven, wellbeing framework based on concepts of positive psychology. This study found that the WoW framework was seen by participants as a practical and effective vehicle for delivering health promotion activities which supported individual and community mental health and wellbeing. Participant stories described changes to health and wellbeing behaviours within the personal lives of those who had participated in WoW training programs, as well as changes to professional practices through the incorporation or wellbeing activities which aimed to strengthen communication and relationships.

Participants expressed confidence in the WoW framework and were utilizing aspects through ‘championing’ the promotion of wellbeing within their community, family, and friendship networks. The concept of ‘champions’ is not new within a community health context, having long been regarded as effective conduit for social change [34]. Schon [35] reported that champions “identify with the idea as their own, and with its promotion as a cause, to a degree that goes far beyond the requirements of their job” (p. 84). WoW champions appeared more aligned with the concept of community health champions (CHCs); an initiative introduced into the British healthcare system [36]. CHCs are typically community workforce members who possess the competence and experience to champion community-driven health promotion initiatives. Through engagement activities they strengthen social connections, effecting healthier and happier communities [36–38]. Participants in this study identified that they regularly went above and beyond their roles to innovate, adapt and deliver WoW activities within their local communities. Numerous examples of how participants had adapted WoW resources for local groups were provided and innovative uses of health promoting materials were identified. The development of a local community of practice ensured that WoW ‘champions’ received ongoing support.

The WoW community of practice promoted the sharing of resources, connections, innovation, and creation of ideas. The community of practice as an avenue for communication, allowed for the implementation of strategic and coordinated WoW events across the Logan and SMBI regions, furthering opportunities to reach more communities and individuals. Clear examples of this ‘ripple effect’ were identified by participants working in the education sector and in neighbourhood centres. More widely, the WoW community of practice continues to be actively cultivated by participants, who genuinely care and seek avenues to actively collaborate, share, think and learn together [33].

Participants in this study identified value in WoW activities being underpinned by the evidence from positive psychology which emphasised the enhancement of mental wellbeing as opposed to more traditional viewpoints for addressing mental illness [39]. Positive psychology theorists suggest that individuals can improve their mental wellbeing through simple and intentional activities such as practicing kindness and expressing gratitude [40]. Numerous examples of activities which supported these elements were identified by participants e.g., random acts of kindness (gifting flowers), gratitude and positive affirmation cards and one-minute ‘masterpiece’ activities. The experience of positive emotions is reported to broaden ones social and psychological resources, in turn assisting the achievement of optimal functioning by building enduring social and psychological resources [41, 42].

The WoW framework was also seen as embracing diversity. Participants identified the effectiveness of activities which brought cultures together by sharing what is common across cultural elements. WoW activities were described as flexible and easily adapted and implemented across a diverse range of cultural settings.

WoW was seen as facilitating the promotion of positive emotions through social inclusion, building and enhancing relationships whilst maintaining a focus on the positive aspects of life. Whilst many participants attended WoW training in relation to their roles as service providers, most reported that training elements and concepts were easily understood and shared with community members. An emphasis on shared responsibility was seen as a strength of the framework. With a focus on developing a sense of achievement, accomplishment and capacity building, the WoW framework was seen as a tool that was accessible and easy to utilise by a range of community members. Through the use of simple wellbeing activities, positive behaviour changes were described, with these changes flowing on to influence the behaviours of family members, friends and community members. Participants saw WoW as having the potential to create progressive, longer-term, ‘ripple’ impacts supporting the enhancement of mental wellbeing of community members.

There are similarities between our study findings and the themes identified in other community-driven, mental wellbeing initiatives, e.g., ‘Our Healthy Clarence’ program [8]. Similarities were noted with the use of a strengths-based approach with a focus on mental wellbeing, increasing hope, communication, inclusivity, and collaboration [8]. However, WoW does not rely on governance and formal structures to succeed but instead relies on community members to take ownership and champion success. This process aligns with community capacity building theories [43]. Whilst the ripple effects that continue beyond participants’ WoW training (e.g., within school and education systems) are promising, further research is needed to appreciate possible longer-term outcomes.

The use of Ripple Effects Mapping (REM) was a strength of this study. REM was found to be well suited to obtaining rich data from a range of participants and was a useful tool in facilitating the collection of previously undocumented and unappreciated ‘ripples’ from the introduction of the WoW framework. REM allowed for the evaluation of program effectiveness through the voices of participants. However, the limitations of the study are acknowledged. The small sample size (*n* = 9) limited the amount of data and the range of stories available for inclusion in the analysis. Participants were recruited from the one urban area and the sample group consisted of largely community-based practitioners. Participants self-selected into the evaluation and as such may not represent the range of experiences of which might be expected from a larger sample. Notably, the voices of those with negative or neutral experiences of WoW may not be represented. In addition, whilst this evaluation collected stories of how the WoW training had influenced participants’ experiences, the outcomes discussed have not been quantitatively measured, and nor can we confidently attribute them wholly to the WoW framework or training program.

## Conclusions

This evaluation indicates that the WoW framework can be used as a tool to enhance the mental wellbeing of individuals and communities. This study highlighted the strengths of using REM to evaluate a health promotion program and provided a base for understanding its effectiveness as captured through the narratives shared by participants. The REM evaluation process assisted service providers and individuals to reflect on, and capture stories of, the use of the WoW framework within their own programs and communities. Feedback from participants supported the premise that a WoW training program has the potential to create a ripple effect, positively contributing toward improved mental wellbeing, enhanced health behaviours, professional practices and connections.

## Data Availability

The data that support the findings of this study are available on request from the corresponding author (VS). The data are not publicly available due to them containing information that could compromise research participant privacy and consent.

## Abbreviations

**WoW**: Wheel of Wellbeing
**QMHC**: Queensland Mental Health Commission
**SMBI**: Southern Moreton Bay Islands
**REM**: Ripple Effects Mapping
**AI**: Appreciative Inquiry
